# A Contribution to a Better Understanding of the *Nitella axillaris* Group (Charales, Charophyceae): A Taxonomic Re-Examination of the *Nitella translucens* Collected in the Province of Ferrara, Italy

**DOI:** 10.3390/plants13213081

**Published:** 2024-11-01

**Authors:** Nadia Abdelahad, Angelo Troia, Klaus van de Weyer, Mauro Iberite, Filippo Piccoli, Michelle T. Casanova

**Affiliations:** 1 Dipartimento di Biologia Ambientale, Sapienza Università di Roma, 00185 Roma, Italy; abdelahadnadia3@gmail.com (N.A.); mauro.iberite@uniroma1.it (M.I.); 2 Dipartimento di Scienze e Tecnologie Biologiche, Chimiche e Farmaceutiche, Università degli Studi di Palermo, 90123 Palermo, Italy; 3 Lanaplan, Lobbericher Str. 5, D-41334 Nettetal, Germany; klaus.vdweyer@lanaplan.de; 4 Dipartimento di Scienze della Vita e Biotecnologie, Università degli Studi di Ferrara, 44121 Ferrara, Italy; fpiccoli3@alice.it; 5 Research Associate, Royal Botanic Gardens Melbourne, Birdwood Ave., South Yarra, VIC 3141, Australia; mt.casanova@federation.edu.au; 6 Natural History Museum, Cromwell Rd, London SW7 5HD, UK

**Keywords:** charophytes, rice field, northern Italy, *Nitella translucens*, *Nitella axillaris*, typification

## Abstract

The identification of a charophyte population found in a rice field in Ferrara, North Italy, collected in 1999 and initially identified as *Nitella translucens*, has been reevaluated. Detailed morphological observations here reported have suggested that the specimen displays characteristics more akin to *Nitella axillaris*, particularly in the structure of its bicellular dactyls and axillary heads. Additional examinations, including scanning electron microscopy (SEM) of oospores and analyses of herbarium specimens—encompassing original materials of *N. axillaris*, *N. axillaris* f. *tenuoir*, and *N. translucens* f. *confervoides*—support this reclassification. Notably, the Ferrara specimens exhibit reticulate oospores and specific morphological traits that align well with *N. axillaris*. However, a syntype of *N. axillaris* housed at the BM Herbarium presents an anomaly, as it features granulate rather than the expected reticulate oospores. This discrepancy indicates a need for further studies, given that the lectotype of *N. axillaris* lacks oospores altogether. For now, the charophyte population from Ferrara can be provisionally assigned to *N. axillaris* “sensu Auctores”. Additionally, it appears to be an alien species introduced into the rice fields of Northern Italy, highlighting the need for further investigation into its taxonomy and distribution.

## 1. Introduction

*Nitella* C. Agardh is one of the most species-rich genera (together with *Chara* L.) in the family Characeae and is also the one exhibiting the highest diversity of vegetative and oospore morphology [1]. It is cosmopolitan and generally occurs in mildly acidic or neutral freshwater environments. Recent morphological and molecular phylogenetic studies have demonstrated the phylogenetic validity of using the external morphology of the oospore wall for diagnosing some species of *Nitella* [1,2,3,4].

In 1999, a *Nitella* population was found in a rice field in the Province of Ferrara (Northern Italy) and ascribed to *Nitella translucens* (Persoon) C. Agardh [5]. Later, while investigating the specimens, it was noticed that some characteristics did not fit with this identification. To be specific, the presence of bicellular dactyls and axillary heads suggests the specimens can be referred to another species, *Nitella axillaris* A. Braun [6].

*Nitella translucens*, described in 1824, has a wide distribution, including in Europe, where it is common in the Atlantic areas [7], but rare elsewhere [8,9]. It is distinguished by being robust, with apparently simple, long, sterile branchlets (that actually furcate at the tips), bicellular dactyls, and gametangia terminal in compact heads [7]. Braun [6] described *Nitella axillaris* in 1859, based on a specimen sent by J. Gollmer, collected in March 1854, “In stagnis prope Caracas”, South America. He compared it to *N. translucens* but distinguished it as a separate species on the basis of its “niedrigen Wuchs und geringere Dicke, mehr genäherte sterile Quirle, starker zugespitzte Endzellen der Segmente der sterile Blätter und etwas kleinere Sporangien unterscheidet (“low stature and smaller thickness, more closely spaced sterile whorls, more pointed terminal cells of the segments of the sterile leaves and somewhat smaller sporangia”) [6]. In 1864, Braun (in Leonhardi [10]) described *Nitella brachyteles*, another similar species, on the basis of material collected in Algeria by Bove in 1839 and 1841. Zaneveld [11] felt that the similarities among *N. translucens, N. axillaris,* and *N. brachyteles* (and two other species, *N. sublucens* and *N. morongii*) might lead them to be considered a single species, and that the name *N. translucens* would have priority, but deferred this change pending examination of the type specimens.

Wood [12,13] essentially followed Zaneveld [11] and subsumed all these species (and more) into *N. translucens* with two subspecies, three varieties, and four forms, essentially equating to previously described species.

In recent years, a different species concept has been applied: Wood’s lumper approach is considered unable to describe the systematic relationships among taxa and has not been widely adopted [14,15] and most of the taxa he subsumed under a broad species concept are now split among several “good species”.

In order to determine the name of the mentioned *Nitella* from Ferrara, in this article new morphological observations on the Italian specimens are presented, including SEM observations of oospores together with other original observations on the types (or original material) of the names of putative taxa (namely: *Nitella axillaris*, *N. brachyteles*, *N. translucens*, and *N. translucens* f. *confervoides*), along with a critical revision of the available information on these taxa.

## 2. Materials and Methods

The doubtful *Nitella* was collected on 9 September 1999 in a rice field in the locality of Le Contane (Convento, Province of Ferrara, Region Emilia-Romagna) [5]. The rice field was flooded at the time of the gathering. The specimens were preserved in 4% formaldehyde and stored at the Department of Life and Biotechnology of the University of Ferrara. They were studied many years after their gathering and were therefore completely discolored.

Morphological observations of those plants were made with a Zeiss stereomicroscope (LM) equipped with a Leica DFC 42 digital camera and the SEM FEI Quanta of the Department of Earth Sciences of the Sapienza University of Rome. In detail, for SEM observations of the oospores, the enveloping cells were removed by needles and by adding 10% Triton X1. The oospores were then sonicated, washed in alcohol, stored at 60 °C all night long, mounted on stubs, and coated with gold. Eighteen oospores were measured, fourteen with LM and four with SEM.

Investigations were made to assess and locate original material and types of the possible candidate species, checking specimens in BM, JE, NY, and PC (herbaria codes according to Thiers [16]): in detail, specimens from JE and NY were requested on loan and specimens in BM and PC were checked on-site (by M.T. Casanova).

The only oospore removed from the JE specimen was prepared according to the method described above. Oospores removed from specimens in BM were soaked in a detergent solution and the enveloping cells were removed with the aid of fine needles. They were dried in alcohol, mounted on aluminium stubs with carbon sticky-tabs, and coated with gold–palladium before viewing with the Zeiss Ultra Plus SEM at BM.

## 3. Results

We present here our new morphological observations on the *Nitella* specimens from Ferrara and then observations and remarks for each of the taxa we have compared with the Italian specimen (listed in the most probable order, the first being the most similar to Italian specimen).

### 3.1. Nitella cf. axillaris from Northern Italian Rice-Fields

**Figure 1:**
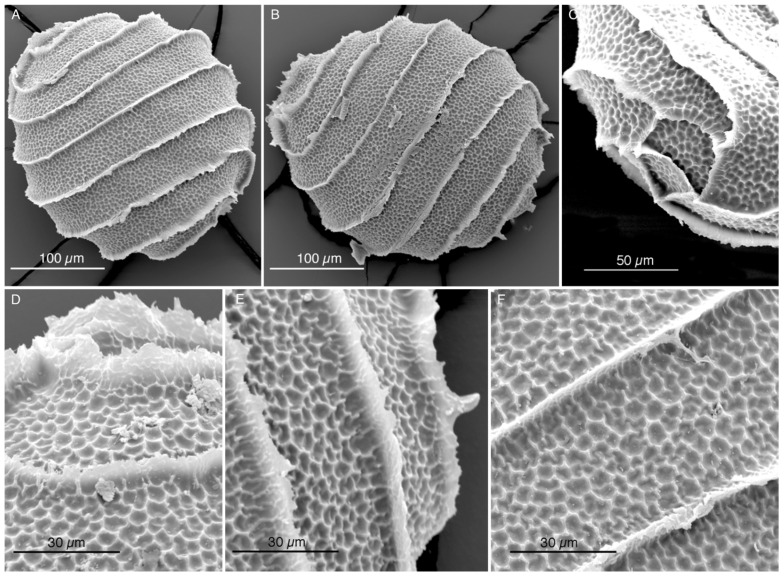
SEM microscope micrograph. Oospores of *Nitella* cf. *axillaris* from the rice fields near Ferrara (Italy) showing a reticulate ornamentation and 7–11 meshes in each fossa. (**A**,**B**). Two whole oospores. (**C**,**D**). Upper poles. (**E**). Lower pole. (**C**–**F**). Details of the fossa walls.

Oospores from the *Nitella* collected in the Italian rice fields of the Province of Ferrara were examined by SEM (Figure 1A–F). They have seven spiral turns and a reticulate ornamentation of the wall. Oospores measures are reported in Table 1. The whole oospores have a mean diameter of 259.98 µm with SEM and 261.06 µm with LM. Each mesh of the fossa has a mean size of 4 µm. The mean width of the fossa is 38 µm.

**Figure 2:**
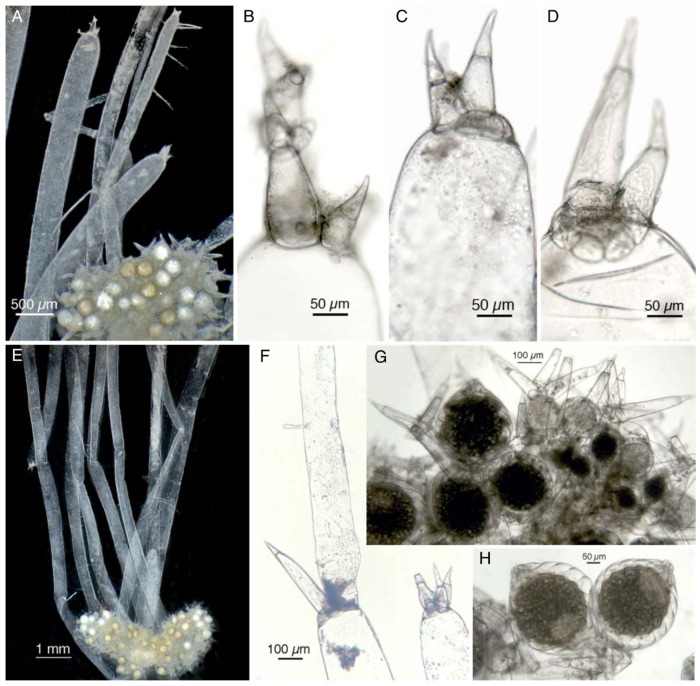
*Nitella* cf. *axillaris* from the rice fields near Ferrara (Italy). (**A**) Stereo-microscope micrograph at the apex of the plant showing an axillary head and sterile branchlets ending with a microscopic crown of bicellular dactyls. (**B**,**D**) Sterile dactyls under the optical microscope. (**B**) Occasional bifid sterile dactyls with rounded protuberances. (**C**,**D**) More or less complete corona of cells seen around the base of sterile dactyls. (**E**) Stereo-microscope micrograph at the center of the plant, showing longer sterile branchlets and two almost elliptic axial heads. (**F**) Branch sprouted from a sterile bicellular dactyl. (**G**) Axial head with bicellular fertile dactyls sometimes two-forked. (**H**) Geminate oogonia.

The morphology of the Italian plants has been revised (Figure 2A–H). Sterile branchlets look prevalently simple (Figure 2A,E), or at most 1 furcate, ending with a rudimentary group of 3–4 bicellular dactyls (Figure 2A–D). Sterile two-celled dactyls are sometimes bifid with rounded protuberances (Figure 2B) and sometimes have sometimes two or a complete corona of cells at the base (Figure 2C,D). Sometimes a branch is observed sprouting from a sterile bicellular dactyl (Figure 2F). Fertile branchlets are numerous, slightly longer than gametangia and masked by them, with generally one furcate, sometimes two-furcated (Figure 2G), compacted into axillary heads, which are elliptic (Figure 2E) and 2–2.9 mm long. Oogonia are more numerous than antheridia and sometimes geminate (Figure 2H), measuring 370–410 µm long and 290–392 µm wide, with 7–8 convolutions. The coronula measures 59 µm to 68.5 µm at the base and is 23 µm high. The diameter of the antheridia ranges from 160 µm to 235 µm.

### 3.2. Notes on Nitella axillaris

*Nitella axillaris* A. Braun, in Monatsber. Königl. Preuss. Akad. Wiss. Berlin 1858: 356 (1859) ≡ *Nitella translucens* var. *axillaris* (A. Braun) R. D. Wood, in Taxon 11: 22 (1962).

TYPE: Orizaba in Mexico, 1853, leg. F. Müller (NY 00887751) (Lectotype designated by R.D.Wood in R.D.Wood & K.Imahori, Monog. Charac. 1: 685. 1965).

Braun [6] clearly identifies as the “reference specimen” of his *N. axillaris* a specimen collected by Gollmer in Venezuela (“in stagnis prope Caracas (im See von Valle) Mart. 1854 legit J. Gollmer (herb. A. Br.)”). After that, he also mentions another specimen collected in 1853 at Orizaba in Mexico by F. Müller, a botanist who died prematurely. According to the Code of Botanical Nomenclature [17], since no holotype has been designated, the two specimens are both syntypes, so the choice of the specimen from Orizaba as the lectotype is legitimate.

Braun did not describe the ornamentation of the oospore’s membrane, but Nordstedt [18] did, and claimed that the oospore was reticulate, and this interpretation was followed by Allen [19] and Groves & Groves [20].

**Figure 3:**
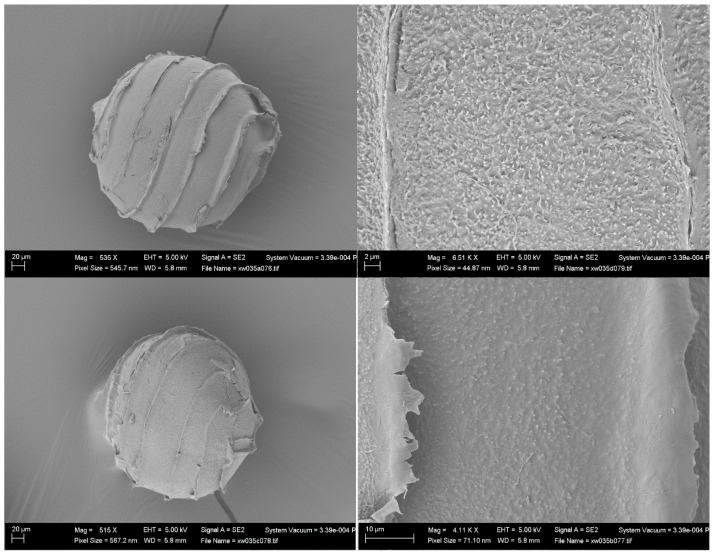
SEM images of an oospore removed by M.T. Casanova from a syntype of *Nitella axillaris* collected in Caracas, Venezuela, and conserved at the NHM of London (BM013828100).

In his description of the taxon (as *N. translucens* var. *axillaris* (A. Braun) R.D. Wood f. *axillaris*), Wood [12] (pp. 683, 685) reported that the oospores have a “reticulate” membrane. Imahori [13] also drew a “perfectly reticulated” oospore membrane for *N. axillaris* (Icon 356, Figure 3). More recently, using both light microscopy and SEM, several authors (e.g., [1,2,21]), observed a reticulated ornamentation of the oospore’s membrane in *N. axillaris* from Japan to Brazil.

To make the scenario more complicated, the membrane of one oospore removed in 2019 by one of the authors of the present contribution (MTC) from a syntype from Caracas conserved at the NHM of London (BM013828100) (with the wording: Ex Museo botanico Berolinensi. *Nitella axillaris* A. Br: “In lagunis prope Valle ad Caracas, March 1854, leg. Gollmer”) has revealed, by SEM, to not be reticulated but granulate with short projections (Figure 3). This observation disagrees with all previous reports for *N. axillaris* (Nordstedt [18], Allen [19], Groves & Groves [20], Wood [12], Imahori [13], Sakayama et al. [2], Borges & Necchi Jr. [1], Ribeiro et al. [21]).

We had the opportunity to also examine the lectotype of *Nitella axillaris* (NY 00887751). It has bicellular dactyls but no oospores, so that—according to the currently available knowledge—it is hard to decide if the name “*Nitella axillaris*” hides two different taxa with different oospores.

**Figure 4:**
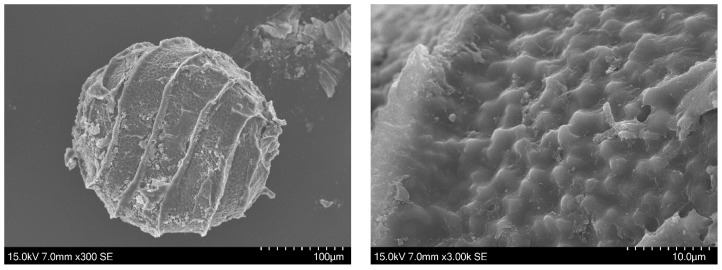
SEM images of an oospore removed by M.T. Casanova from the herbarium specimen PC0610495 (Spruce 6551-2) (“*Nitella axillaris* f. *tenuior*”).

**Figure 5:**
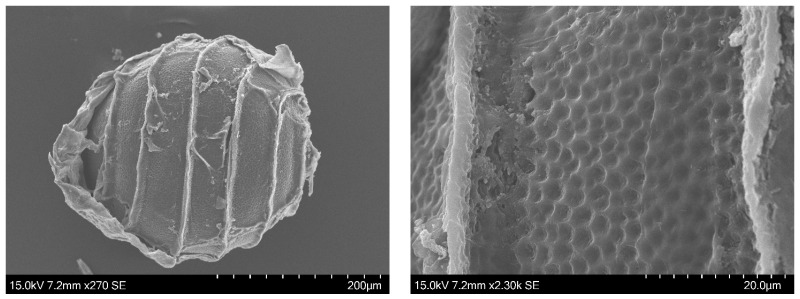
SEM images of an oospore removed by M.T. Casanova from the herbarium specimen PC0610594 (Sintensis 6618) (“*Nitella axillaris* f. *tenuior*”).

Two additional specimens from South America, under the name *Nitella axillaris* f. *tenuior* (that seems to be a “nomen nudum”), have been examined (PC0610495 Chanduy in litore Maris Pacifici, Spruce 6551-2; and PC0610594 Manati, Apr. 1889, P. Sintensis 6618) (Figure 4 and Figure 5, respectively). Oospores from those specimens, removed and observed by one of the authors (MTC), were revealed to have nodulate ornamentation in one case (Figure 4) and reticulate ornamentation in the other case (Figure 5).

### 3.3. Notes on Nitella brachyteles

*Nitella brachyteles* A. Braun, in Verh. Naturf. Vereins Brünn Abh. 2: 173 (1864). 

TYPE: not yet designated (a lectotype will be designated in [22]).

**Figure 6:**
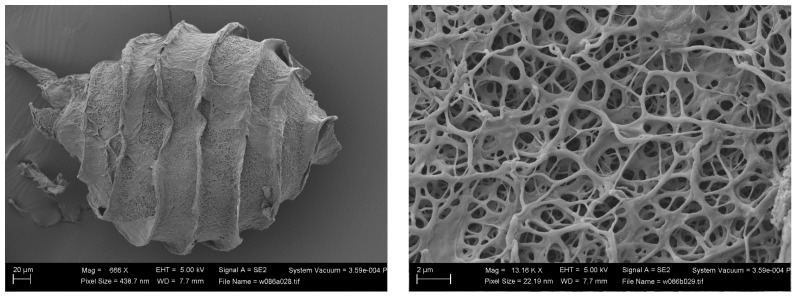
SEM images of an oospore of *Nitella brachyteles* removed by M.T. Casanova from the herbarium specimen BM013828170: Bové, Nicolas s.n. 1 6 1839 Lake Ouberia (Houberia), La Calle. [22].

Zaneveld [11] (p.70), and other authors after him, considered *Nitella brachyteles* A. Braun as variety of *N. translucens*. Unfortunately, we do not know the oospore membrane ornamentation of the lectotype [22]. The only SEM images of *N. brachyteles* oospore we have seen are those made by M.T. Casanova (Figure 6): they show a fibrous membrane, different from that of the *Nitella* of the Italian rice field.

### 3.4. Notes on Nitella translucens

*Nitella translucens* (Pers.) C. Agardh, Syst. Alg.: 124 (1824).

TYPE: Eaton Pool, near Shrewsbury [England], E. Williams, 1800; LINN; HS1432-6-1 (Neotype, Wood & Imahori 1965: 679).

**Figure 7:**
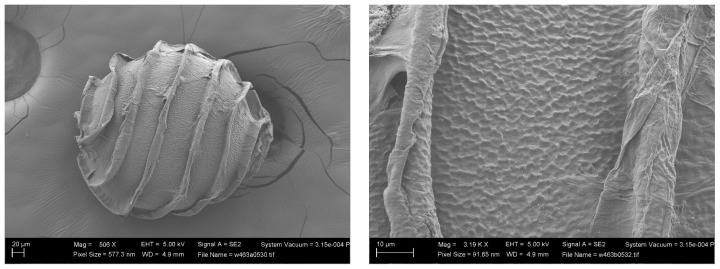
SEM images of an oospore of *Nitella translucens* from the herbarium specimen BM013828852 (Vise, Normandy) [7] (photo by M.T. Casanova).

The wall ornamentation of the oospores of the type material of *N. translucens* remains unknown [7], but John and Moore [23] provided SEM images of the oospore wall of an Irish specimen identified as *N. translucens*. The oospore wall of this specimen came from the Bullock-Webster slide collection. It appears to have a “minutely or finely” reticulate pattern with “surface wrinkles” that “run almost parallel to the long axis of the spore” [23]. A similar pattern was also observed by one of the authors (MTC) in a specimen collected by Pelvet at Vise, Normandy, and kept in London (BM013828852) (Figure 7) [7]. According to John and Moore [23], there is a stability “between different collections of the same taxon, sometimes from different continents”; and they gave as example the American material of *N. translucens*, which has an oospore ornamentation “identical to that found on the oospores of French and Irish specimens” that they had examined.

### 3.5. Notes on Nitella translucens f. confervoides

*Nitella translucens* f. *confervoides* Thuill. ex Migula, Charac. Deutschl.: 146 (1890).

TYPE: Locus classicus: “Mit der typischen *N. translucens* zusammen bei Köln (Odenthal)” (“With the typical *N. translucens* together near Cologne (Odenthal)”).

**Figure 8:**
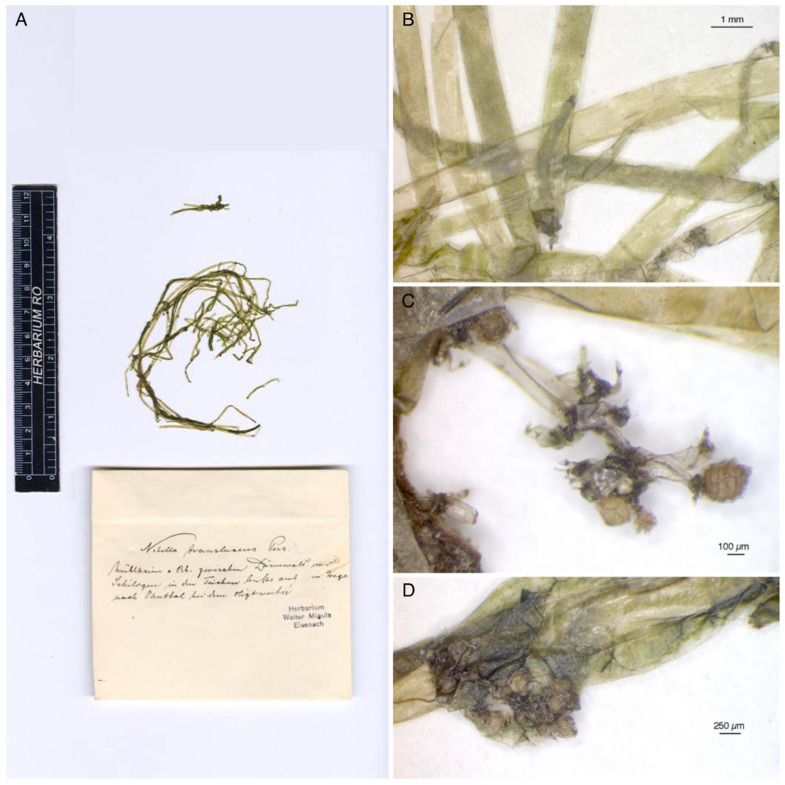
Migula’s original material of *Nitella translucens* f. *confervoides* conserved at JE (**A**). The specimen with its label (*Nitella translucens* Pers.). (**B**) The measures of the axes correspond to those described by Migula for *N. translucens* f. *confervoides*. (**C**) Micrograph of the only mature oospore found in Migula’s material. (**D**) Other non-mature gametangia.

*N. translucens* f. *confervoides* is closely related to *N. brachyteles* [24] and was probably correctly described for the first time by Migula [25]. We studied the original material regarding this name. Migula used the name *Nitella translucens* f. *confervoides* in his book [25] (p. 146), but the label of the original material bears only the binomial *N. translucens* Pers., without further notes (Figure 8A). However, the thickness of the axes of this material (0.740 mm-0.926 mm) (Figure 8A,B) corresponds to that described by Migula for the f. *confervoides* (“der Stengel wird nur 0.75 bis höchstens 1 mm dick”).

**Figure 9:**
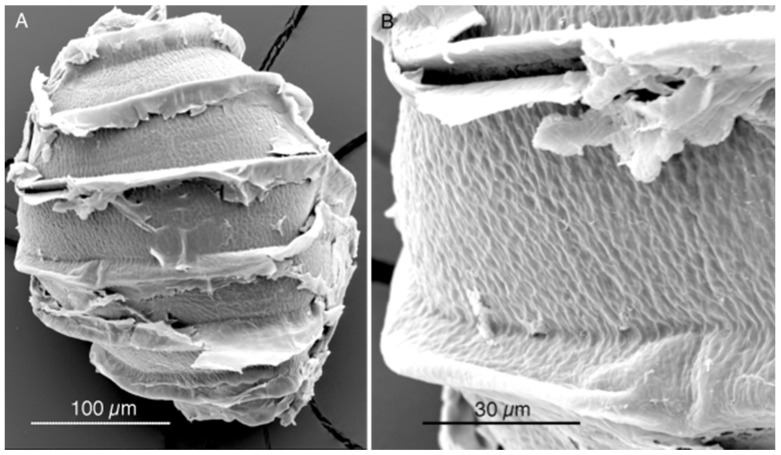
SEM micrographs of an oospore from Migula’s original material of *Nitella translucens* f. *confervoides* showing a fine reticulum oriented parallel to the long axis of the oospore. (**A**). Whole oospore with still residues of the enveloping cells. (**B**) Detail of a fossa.

Only one ripe oospore, 298 µm long and 254 µm wide, was found in Migula’s original material (Figure 8C,D). It was observed with SEM. This oospore appears to have a fine reticulum oriented parallel to the long axis of the oospore (Figure 9A,B), an ornamentation different from that of the *Nitella* of the Italian rice field (see Section 3.1 and Section 4).

## 4. Discussion and Conclusions

The oospore walls of the *Nitella* from Ferrara have a reticulated aspect (Figure 1A–F). Comparing them to the oospores of the other similar taxa that we have here investigated, they do not correspond to *N. translucens*, nor to *N. translucens* f. *confervoides*, nor to *N. brachyteles*, as we have seen above. On the contrary, the only species of this group with a reticulate oospore is *N. axillaris*.

All the authors, from Nordstedt [18] onwards (Allen [19], Groves & Groves [20], Groves & Bullock-Webster [26], Wood & Imahori [12,13], Sakayama et al. [2], Borges and Necchi Jr. [1], and Ribeiro et al. [21]) reported a “reticulate” ornamentation for the oospores of *N. axillaris*. Here we presented an additional image supporting this view (Figure 5), showing a reticulate oospore for *N. axillaris* f. *tenuior* (even though the reticulate pattern in this last taxon appears different compared to the pattern of the Italian specimen, as shown in Figure 1). Therefore, according to these observations, the *Nitella* of the north Italian rice-field is attributable to *N. axillaris*.

Since, however, as shown above, the syntype of *N. axillaris* conserved at London (BM013828100) has oospores not reticulate, but granulate with short projections (Figure 3), and the lectotype of *N. axillaris* (NY 00887751) has no oospores at all, further investigations are needed to assess if *N. axillaris* has reticulate or granulate oospores and, if granulate, to ascertain the name of the taxon with reticulate oospores.

The morphology of the Italian plants shares a certain number of characteristics with *N. translucens* var. *axillaris* f. *caroliniana* K. Imahori & R.D. Wood (=*N. caroliniana* [K. Imahori & R.D. Wood] R.D. Wood) through its occasionally bifid sterile dactyls and the more or less complete corona of cells around the base of some sterile dactyls (Figure 2). Though preliminary phylogenetic data suggests that *N. caroliniana* may represent a distinct taxon separate from *N. axillaris* (Kenneth G. Karol, pers. comm.), fresh collections of the *Nitella* from the Italian rice field and further investigations will be needed to assess its phylogenetic affinity.

An oospore with very similar ornamentation to that of the Italian *Nitella* (i.e., reticulate) is the lectotype of *Nitella sphaerocephala* J. Groves preserved at the NHM of London (BM013821252). But the morphology of this species does not correspond to the *Nitella* from the Italian rice field: the branchlets do not end with a rudimental whorl of bicellular dactyls but are instead mostly three-celled and aligned, the oospores are larger, the coronula higher, the sterile branchlets more forked, and the fertile heads more roundish [12,27].

So, according to the available data and to our observations, the *Nitella* collected in the rice fields near Ferrara has to be provisionally referred to as *Nitella axillaris* “sensu Auctores”, and its features—in our opinion—deserve to be published as a contribution to the taxonomy (and probably also the phylogeny, biogeography and ecology) of this problematic group of species. A precise identification of the Italian plants, possible only in the framework of a global review of the group, will allow confirmation of if—as it seems probable—the specimens collected near Ferrara belong to an alien species.

## Figures and Tables

**Table 1:** Measures of oospores, without the enveloping cells, from the *Nitella* collected in the Ferrara rice fields.

Measurements	Min. (µm)	Max. (µm)	Mean (µm)	SE (µm)	N
Whole oospores length (SEM)	249.29	291.10	259.98	10.38	4
Whole oospores length (LM, without enveloping cells)	240.27	278.15	261.06	3.40	14
Meshes (dimensions) (SEM)	1.17	7.12	4.04	0.27	41
Width of the fossa (SEM)	28.13	49.15	37.93	2.50	14

## Data Availability

Data are contained within the article.
